# Clinical Disease and Outcomes of Group B Streptococcus Bloodstream Infections at a Teaching Hospital in Saudi Arabia From 2015 to 2022

**DOI:** 10.7759/cureus.54442

**Published:** 2024-02-19

**Authors:** Nisreen Bajnaid, Reham Kaki

**Affiliations:** 1 Department of Medicine, King Abdulaziz University, Jeddah, SAU; 2 Department of Infectious Diseases, King Abdulaziz University, Jeddah, SAU; 3 Internal Medicine, Infectious Disease & Infection Control, King Abdulaziz University Hospital, Jeddah, SAU

**Keywords:** skin and soft tissue infection, mortality, methicillin-resistant staphylococcus aureus, invasive disease, group b streptococcus, bacteremia

## Abstract

Background

Invasive disease due to group B *Streptococcus* (GBS) infection in adult males and nonpregnant females can cause various diseases, such as primary bacteremia, endocarditis, skin and soft tissue infection (SSTI), and meningitis. Especially in older people, invasive GBS infection has a high case fatality rate. In Saudi Arabia, little is known about the clinical signs and symptoms of GBS bacteremia and the associated risk factors and mortality rate.

Methodology

We performed a retrospective study at King Abdulaziz University Hospital in Jeddah, Saudi Arabia, a large tertiary hospital, to investigate clinical disease, potential risk factors, susceptibility patterns, and mortality related to GBS in adult males and nonpregnant females diagnosed with GBS bacteremia. All patients ≥14 years of age with GBS-positive blood cultures from January 1, 2015, until December 31, 2022, were included. Patient data such as age, sex, comorbidities, hospital ward, length of hospital stay, monomicrobial versus polymicrobial bloodstream infection, antimicrobials used for treatment, complications, whether an infectious disease specialist had seen them, and outcomes were extracted from the electronic health records.

Results

A total of 50 patients with GBS bacteremia met the inclusion criteria. The mean age of these patients was 57.0 years (SD = 16.0), and 27 (54%) were female. The 90-day mortality was 11 (22%). In total, 34 (68%) patients had a monomicrobial infection, and among those with polymicrobial infection, methicillin-resistant *Staphylococcus aureus* was the most common co-infection (56%, n = 9/16). The most common source of infection was SSTI and wound infection in 24 (48%) patients. Most patients had one or more comorbidities; the mean Charlson comorbidity index was 3.8 (SD = 2.4). The most prevalent comorbidity was diabetes mellitus in 35 (70%) patients. Of all variables analyzed, only age was significantly associated with mortality (p = 0.016), and age had a predictive value for mortality (p = 0.035).

Conclusions

In Saudi Arabia, as in other countries, GBS is an important pathogen, especially in older people, that should be considered when encountering a patient with bacteremia. In addition, in patients over 65 years old, GBS bacteremia carries a high risk for mortality.

## Introduction

Group B *Streptococcus* (GBS), also known as *Streptococcus agalactiae*, is a gram-positive bacterium that causes invasive infections in pregnant females, neonates, and adults aged 65 and older [1,2]. Especially in the older age group, invasive GBS infection can cause a range of diseases with high case fatality [3]. In several studies, the main risk factors for invasive disease in adult males and nonpregnant females were reported to be older age, diabetes, liver disease, immunosuppression, neurological disease, and human immunodeficiency virus [4-6].

Invasive diseases due to GBS infection include primary bacteremia, endocarditis, skin and soft tissue infection (SSTI), osteomyelitis, meningitis, and urinary tract infection (UTI) [3,7]. GBS bacteremia can also be secondary to other focuses, such as central line infections, as was the case in a catheter-related outbreak in hemodialysis patients [8]. It can also seed to other organs and cause diseases that are difficult to treat, such as infective endocarditis and osteomyelitis [9].

This study aimed to investigate clinical presentations, outcomes, and mortality of invasive disease, particularly bacteremia, caused by GBS in nonpregnant adults in Saudi Arabia, as the information about this particular organism in this region is very limited.

## Materials and methods

Study design and patient selection

We performed a retrospective study at King Abdulaziz University Hospital in Jeddah, Saudi Arabia, a 1,000-bed tertiary teaching hospital. The study investigated clinical disease, potential risk factors, and mortality related to GBS. Using digital hospital records, we identified all patients ≥14 years of age with GBS-positive blood cultures from all clinical departments except obstetrics and pediatrics from January 1, 2015, until December 31, 2022. We excluded patients <14 years old and pregnant females. The study was approved by the hospital’s ethical review committee (Unit of Biomedical Ethics, Research Ethics Committee) of King Abdulaziz University in Jeddah, Saudi Arabia (approval number: 222-23).

Data collection

Patient information, such as age, sex, comorbidities, clinical ward type, length of hospital stay, monomicrobial versus polymicrobial bloodstream infection, antimicrobials used for treatment, complications, whether an infectious disease specialist had seen them, and outcomes were extracted from the electronic health records. Clearance was assessed by repeat blood cultures. The time to clearance was calculated between the first positive and the subsequent negative culture following treatment. The Charlson comorbidity index was calculated based on the data in the patients’ health records. As outcomes, we analyzed the length of hospital stay, time to infection clearance, and all-cause 90-day mortality.

Identification of bacteria

Bacterial isolates were grown for 24 hours on blood, MacConkey, and Sabouraud agar plates and then identified with reference to the Clinical and Laboratory Standards Institute (CLSI) criteria [10,11]. The minimal inhibitory concentration (MIC) was measured using the CLSI criteria [10,11]. VITEK 2 compact (bioMérieux, France) identified the isolates, and matrix-assisted laser desorption ionization-time of flight mass spectrometry (bioMérieux, France) was used for further confirmation. No molecular or molecular typing was performed on any of the isolates.

Statistical analysis

This retrospective chart review study included both categorical and continuous variables. Categorical variables were presented as frequencies and percentages, while the numerical variables were presented as median, mean, and standard deviation. Numerical data were checked for normality using the Kolmogorov-Smirnov and the Shapiro-Wilk tests. Both tests revealed non-normal distributions. Therefore, nonparametric tests were performed to analyze the data. Chi-square tests analyzed categorical variables to assess associations. The Spearman correlation test was performed to assess the correlation between numerical variables. The Mann-Whitney U test assessed relationships between categorical and continuous variables. Finally, a binary logistic regression test identified the factors predictive of mortality in GBS cases. The data were analyzed and presented as odds ratio at a 95% confidence interval using SPSS version 24.0 (IBM Corp., Armonk, NY, USA).

## Results

Patients’ demographics and clinical characteristics

We identified 50 patients with GBS bacteremia who met the inclusion criteria. Six pregnant/postpartum females (10.7% of cases) with GBS bacteremia were excluded. The median age of the 50 patients was 59.5 years (mean = 57.0 years, SD = 16.0), ranging from 27 to 84 years, and 27 (54%) were female (Table 1). The 90-day mortality was 11 (22%).

**Table 1:** Distribution of the 50 cases by gender, age group, type of infection, duration of therapy, location in hospital, and mortality group. MICU: medical intensive care unit; SICU: surgical intensive care unit; n: number

Variables	Attributes	n	%
Gender	Male	23	46.0
	Female	27	54.0
Age group	<65 years	32	64.0
	≥65 years	18	36.0
Type of infection	Monomicrobial	34	68.0
	Polymicrobial	16	32.0
Duration of therapy	<7 days	10	20.0
	≥7 days	40	80.0
Location in hospital	Emergency	25	50.0
	Medical floor	14	28.0
	MICU	4	8.0
	SICU	3	6.0
	Surgery floor	4	8.0
Mortality group	<30 days	10	20.0
	<90 days	1	2.0

**Figure 1:**
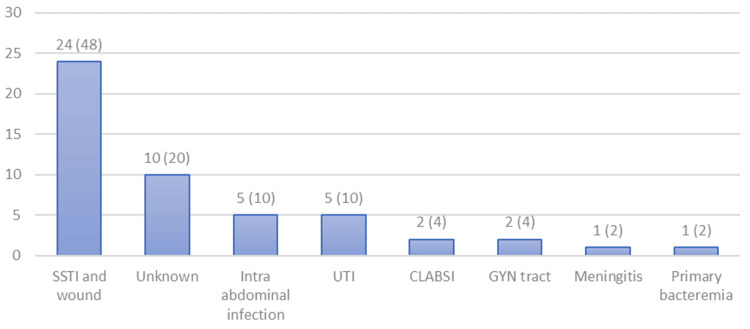
Number and percentage of the sources of the GBS infection. Primary bacteremia is defined as a case of bacteremia where no source was identified despite a complete workup. The bacteremia source was defined as unknown when an incomplete workup was done to identify the source. CLABSI: central line-associated bloodstream infection; GYN: gynecology; SSTI: skin and soft tissue infection; UTI: urinary tract infection

The most common source of GBS infection was SSTI and wound infection in 24 (48%) patients, and the least common sources were meningitis (n = 1, 2%) and primary bacteremia (n = 1, 2%) (Figure 1). Among the 24 SSTI cases, 12 had complicated SSTI (four necrotizing fascitis, eight diabetic foot infection), and the other 12 had uncomplicated SSTI (namely cellulitis).

**Figure 2:**
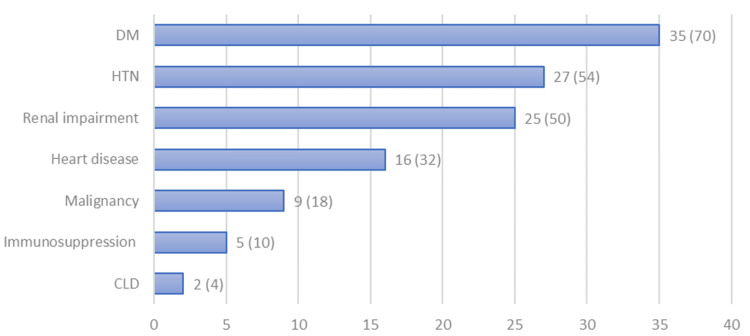
Number and percentage of comorbid factors. CLD: chronic liver disease; DM: diabetes mellitus; HTN: hypertension

The most prevalent comorbid factor was diabetes mellitus in 35 (70%) patients, followed by essential hypertension observed in 27 (54%) patients (Figure 2). The resulting mean Charlson comorbidity index was 3.8 (SD = 2.4) (Table 2). All patients were hospitalized, with 25 (50%) patients admitted to the emergency department. The median length of hospital stay was 15 days, ranging from 1 to 95 days (Table 2).

**Table 2:** Distribution of all continuous variables. SD: standard deviation

Variables	Mean	Median	SD	Range
Age, in years	57.5	59.5	16.0	27–84
Length of hospital stay, in days	62.8	15	306.3	1–95
Time to clearance, in days	5.1	4	3.8	1–12
Charlson comorbidity index	3.8	4	2.4	0–10
Duration of antibiotic treatment, in days	17.6	14	17.3	1–90

Infection specifics

Of the patients with GBS bloodstream infection, 34 (68%) had a monomicrobial infection (Table 1). Among the 16 polymicrobial infection cases, nine (56%) had a co-infection with methicillin-resistant *Staphylococcus aureus*, one (6.3%) with *Klebsiella*, one (6.3%) with both *Klebsiella *and *Pseudomonas*, and for five patients the pathogen was not specified. All GBS strains were fully susceptible to penicillin and clindamycin, and no other susceptibility testing was recommended for GBS.

**Figure 3:**
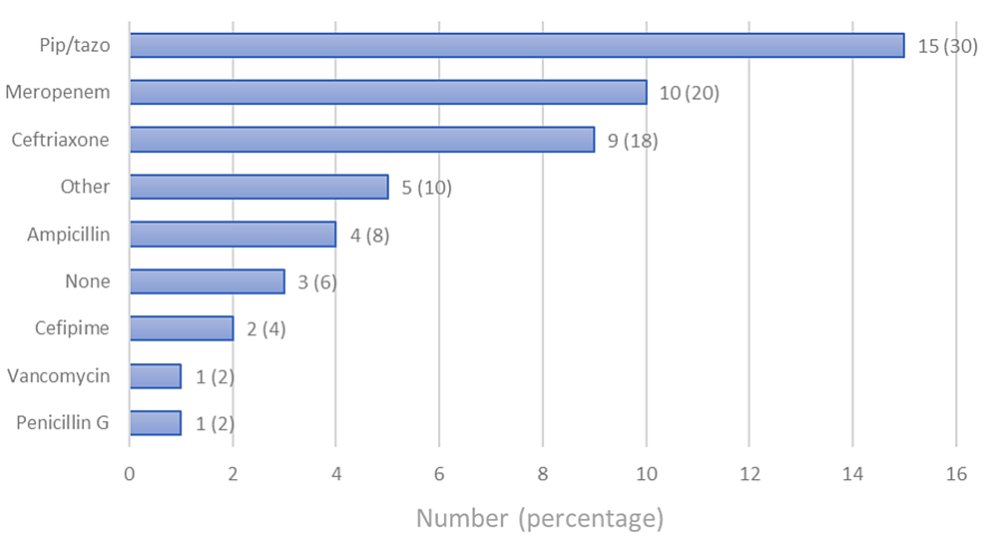
Type of antibiotics used to treat the patients. Pip/Tazo: a combination of piperacillin and tazobactam

As their initial antibiotic therapy, the majority (n = 46, 92%) of the patients received a β-lactam (piperacillin and tazobactam, meropenem, ampicillin, cefepime, or penicillin G) and 21 (42%) received vancomycin. The most used antibiotic was a combination of piperacillin and tazobactam, which was given to 15 (30%) patients, followed by meropenem given to 10 (20%) patients. Only one patient received vancomycin (Figure 3), who developed a vancomycin allergy. The antibiotic “other” in Figure 3 indicates cases where a second antibiotic was given for a concomitant infection, and “none” indicates cases where no antibiotics were given to patients who were discharged before the blood culture result was known or the primary physician chose not to give antibiotics as they thought the GBS in the blood was a contaminant. The median duration of antibiotic therapy was 14 days, ranging from 1 to 90 days (Table 2). Most patients were seen by an infectious disease specialist (n = 36, 72%).

The correlation between age, length of hospital stay, time to clearance, Charlson comorbidity index, and duration of antibiotic treatment was analyzed and, unsurprisingly, a positive correlation was found between age and Charlson comorbidity index (r = 0.351, p = 0.012 (Table 3).

**Table 3:** Correlation between numerical variables. LOS: length of stay; NA: not applicable

Variables	Correlation	LOS	Time to clearance	Charlson comorbidity index
Age	r	-0.038	0.098	0.351
	P-value	0.794	0.581	0.012
LOS	r	NA	0.124	0.198
	P-value	NA	0.486	0.167
Time to clearance	r	NA	NA	0.204
	P-value	NA	NA	0.247
Charlson comorbidity index	r	NA	NA	NA
	P-value	NA	NA	NA
Duration of antibiotic treatment	r	NA	NA	0.179
	P-value	NA	NA	0.215

Association of variables with mortality

The relationship between mortality and the categorical variables gender, type of infection, and whether an infectious disease specialist saw the patient was assessed. None of these variables were associated with mortality (Table 4).

**Table 4:** Association between mortality and the categorical variables gender, type of infection, and seen by an infectious disease specialist.

Variables	Attributes	Mortality = Yes	Mortality = No	P-value	Odds ratio
Gender	Male	6 (26.1)	17 (73.9)	0.520	0.644
	Female	5 (18.5)	22 (81.5)		
Type of infection	Monomicrobial	8 (23.5)	26 (76.5)	0.704	0.750
	Polymicrobial	3 (18.8)	13 (81.3)		
Seen by an infectious disease specialist	Yes	6 (16.7)	30 (83.3)	0.144	0.360
	No	5 (35.7)	9 (64.3)		

The relationship between mortality and the continuous variables age, length of hospital stay, time to clearance, Charlson comorbidity index, and duration of antibiotic treatment was also assessed. The patient’s age was found to be significantly associated with mortality (p = 0.016) (Table 5).

**Table 5:** Relationship between mortality and the continuous variables age, length of hospital stay, time to clearance, Charlson comorbidity index, and duration of antibiotic treatment. SD: standard deviation

Variables	Mortality = Yes	Mortality = No	P-value
Age, in years ± SD	67.27 ± 13.70	54.72 ± 15.68	0.016
Length of hospital stay, in days ± SD	15.36 ± 9.94	76.23 ± 346.58	0.535
Time to clearance, in days ± SD	5.00 ± 3.34	5.08 ± 3.93	0.951
Charlson comorbidity index ± SD	4.82 ± 2.32	3.51 ± 2.35	0.118
Duration of antibiotic treatment, in days ± SD	13.27 ± 8.66	18.77 ± 18.95	0.916

Factors predicting mortality

A binary logistic regression analysis was performed to identify the factors predictive of mortality. The variables age, length of stay, diabetes mellitus, hypertension, renal impairment, heart disease, chronic liver disease, malignancy, type of infection (monomicrobial or polymicrobial), and whether or not seen by an infectious disease specialist were included in the analysis. The overall model prediction was 84%. The model revealed that the patient’s age was a statistically significant predictor of mortality, with a p-value of 0.035 and an odds ratio of 1.087 (95% confidence interval = 1.006-1.174) (Table 6). All specific information about the 11 patients who died is shown in Table 7.

**Table 6:** Factors predictive of mortality. *: Based on binary logistic regression analysis.

Factors	P-value*	Odds ratio	95% confidence interval	
			Lower	Upper
Age	0.035	1.087	1.006	1.174
Length of hospital stay	0.892	0.995	0.918	1.077
Diabetes mellitus	0.267	4.491	0.316	63.834
Hypertension	0.652	1.774	0.146	21.475
Renal impairment	0.090	0.083	0.005	1.469
Heart disease	0.165	0.210	0.023	1.901
Chronic liver disease	0.968	1.074	0.031	37.010
Malignancy	0.948	1.136	0.025	50.816
Type of infection (monomicrobial vs. polymicrobial)	0.670	0.623	0.071	5.494
Seen by an infectious disease specialist	0.242	3.805	0.406	35.620

**Table 7:** Demographic and clinical details of the 11 patients who died. CCI: Charlson comorbidity index; CLABSI: central line-associated bloodstream infection; DM: diabetes mellitus; GBS: group B *Streptococcus*; F: female; LOS: length of stay; M: male; NA: not analyzed; SSTI: skin and soft tissue infection; UTI: urinary tract infection

Sex	Age	LOS	Time to clearance	Source of GBS bacteremia	Type of infection	Co-infection detected	Comorbidities	CCI	Antibiotics used	Duration of antibiotic treatment	Mortality	Culture to death	Cause of death
F	33	1	NA	CLABSI	Polymicrobial	*P. aeruginosa*, *K. pneumoniae*	Chronic kidney disease, ischemic heart disease	2	Cefepime	1 day	<30 days	1 day	Septic shock
M	69	22	1 day	Peritonitis	Monomicrobial	None	DM, hypertension, chronic kidney disease, ischemic heart disease	4	Piperacillin-tazobactam	22 days	<30 days	22 days	GBS sepsis
F	80	16	4 days	Unknown	Monomicrobial	None	DM, hypertension, chronic kidney disease, ischemic heart disease, chronic liver disease	10	Piperacillin-tazobactam	16 days	<90 days	83 days	Sepsis due to DM foot
F	65	2	NA	Unknown	Monomicrobial	None	DM, chronic kidney disease	3	Meropenem	2 days	<30 days	1 day	GBS sepsis
M	75	11	1 day	SSTI and wound	Monomicrobial	None	DM, hypertension, chronic kidney disease, ischemic heart disease	5	Piperacillin-tazobactam	11 days	<30 days	10 days	GBS sepsis
M	71	28	4 days	Intra-abdominal infection	Polymicrobial	Methicillin-susceptible *S. aureus*	Malignancy cholangiocarcinoma	6	Piperacillin-tazobactam	17 days	<30 days	28 days	End-stage cholangiocarcinoma
M	83	11	10 days	UTI	Monomicrobial	None	DM, hypertension, chronic kidney disease, ischemic heart disease	4	Penicillin G	14 days	<30 days	10 days	Acute ischemic heart disease
F	64	15	8 days	Meningitis	Monomicrobial	None	DM, chronic kidney disease, ischemic heart disease	5	Ceftriaxone	15 days	<30 days	14 days	Meningitis
F	78	8	NA	SSTI and wound	Monomicrobial	None	Hypertension	2	Piperacillin tazobactam	4 days	<30 days	2 days	Acute pulmonary embolism
M	59	30	8 days	CLABSI	Polymicrobial	Methicillin-susceptible *S. aureus*	DM, hypertension, chronic kidney disease	5	Meropenem and vancomycin	30 days	<30 days	20 days	Infective endocarditis
M	63	25	4 days	SSTI and wound	Monomicrobial	None	DM, hypertension, chronic kidney disease, ischemic heart disease,	7	Meropenem	14 days	<30 days	23 days	Congestive heart failure

## Discussion

This study highlights the fact that in Saudi Arabia GBS disease is probably an uncommon disease in nonpregnant adults, as we only found 50 cases of GBS bacteremia over an eight-year period at a large tertiary hospital.

Most of our patients had one or more comorbidities such as diabetes, hypertension, renal impairment, heart disease, chronic liver disease, and malignancy. These comorbidities are in line with the known most common risk factors: obesity in 54% and diabetes in 53%, which were reported in a large study of invasive GBS infection in the United States [6], and diabetes in 37%, obesity in 35%, and renal disease in 21% in a study in Belgium [12]. Comorbidities can lower the immune response and make the individual more susceptible to GBS infection. This is, for instance, seen in diabetes mellitus patients as they have chemotaxis and phagocytosis impairment, which makes them more susceptible to many infections, including GBS. Unfortunately, as body mass index was not analyzed in our study, we cannot confirm whether obesity was a risk factor in our patients.

Most of our patients had a secondary source of bacteremia, and the most common source of that was SSTI, which accounted for 48% of the cases, followed by primary bacteremia in 20% and UTI in 12%. These percentages were similar to those in a study of 126 patients in the United States that noted primary bacteremia accounted for 12.9% of cases [13]. The same study reported a mortality rate of 13.4% due to invasive GBS bacteremia [13], which was somewhat lower than the 90-day mortality rate of 22% in our patients. Age was the most important risk factor in predicting mortality in our patients. This finding echoes several studies that showed that the incidence of invasive GBS doubles in patients who are 65 years or older when compared to 50-64-year-old patients [6,14-20]. The overall 90-day mortality rate in our GBS bacteremia patients was around 22%. In other studies, mainly 30-day mortality was reported, which ranged from 6.5% to 14.4%, while several of these studies also included patients without bacteremia [6,21,22], which may have reduced mortality. In a study that analyzed 12-month mortality in patients with invasive GBS infections, the mortality was 29% [23]. Potential contributing factors to the high 90-day mortality in our patients may have been polymicrobial infection, late presentation, higher prevalence of diabetes, advanced age, as well as the presence of other comorbidities such as chronic kidney disease and ischemic heart disease.

Our patients with SSTI had SSTI either in the form of cellulitis, diabetic foot, or surgical site infection. One group noted, based on a case and a review of other cases in the literature, that recurrent GBS cellulitis was mainly seen in patients with chronic lymphedema [24]. In contrast, none of our SSTI patients had lymphedema or the presence of a foreign body. We also did not observe patients with severe SSTI in the form of necrotizing fasciitis. This finding was similar to reports in which necrotizing fasciitis was only an occasional finding, which suggests that GBS commonly causes SSTI but not necrotizing fasciitis [25,26].

Only one patient in our study, a 64-year-old woman, had meningitis; her symptoms were very severe, and she died. A review reported that in adults with GBS meningitis, an overwhelming infection often presented with features of septic shock and that these patients had poor outcomes as the case fatality rate was 34%. Among the survivors, 7% had neurological sequelae, which in these cases presented as deafness [27]. Some studies reported that 5.6-7.3% of patients had recurrent episodes of GBS bacteremia with a mean interval of 24 weeks [6,9]; we, however, did not see any patient with recurrent bacteremia in the eight years covered by our study.

The majority of the patients in our study had monomicrobial infections, while 32% had a polymicrobial infection, in which *Staphylococcus aureus* was the most common pathogen, followed by *Klebsiella*. In a small retrospective case review study from June 2010 to October 2011 in a hospital in Malaysia, 45% of the 18 cases had a polymicrobial infection, with *Staphylococcus aureus* being the most common concurrent bacterial isolate [28]. In a larger study in Taiwan that included 94 patients, *Staphylococcus aureus* and *Klebsiella pneumoniae* were also the most common pathogens [21].

Whether or not an infectious disease specialist was consulted for a patient with GBS bacteremia did not affect mortality in our study. A study that specifically examined the impact of infectious disease consultation in the setting of bacteremia showed that infectious disease consultation led to a significantly lower hazard of death (p < 0.05) [29]. The difference between their and our findings may be that the GBS strains isolated in our study were susceptible to the antibiotics commonly used in bacteremia, such as β-lactams and vancomycin, while Tang and colleagues looked at bacteremia cases caused by a range of bacteria of which antibiotic susceptibilities were not tested [29]; hence, it is unknown whether the antibiotics used when no infectious disease specialist was consulted were appropriate.

This study has several limitations. First, there was a small number of patients, as we only found a limited number of patients 14 years or older. Second is the nature of the study, which is a retrospective study as this is a rare infection which limits the utilization of the identified mortality risk factors using odds ratio. A prospective study design would be better, but it would take a very long time to collect a reasonable number of patients to study. Third, this is a single-center study, and it would be better to perform a multicenter study to have more robust data. Lastly, none of the isolates were typed as our center was not able to perform typing. It would have been interesting to obtain typing data to better understand which GBS types are more invasive or associated with higher mortality so that treatment can be adjusted accordingly. The strength of the study is that it is the first study from Saudi Arabia investigating GBS infection in nonpregnant adults performed at a large tertiary teaching hospital.

## Conclusions

The study highlighted that in Saudi Arabia, GBS is an important pathogen, especially in older people, that should be considered when faced with a patient with bacteremia. In this study, age was the most important risk factor in predicting mortality in our patients. We also found that in patients with GBS bacteremia, polymicrobial infection is not uncommon and is mostly associated with *Staphylococcus aureus*. The patients who died had a higher Charlson comorbidity index than those who survived, although this difference was not statistically significant.
